# Acute myeloid leukemia of donor origin after allogeneic stem cell transplantation from a sibling who harbors germline *XPD *and *XRCC3 *homozygous polymorphisms

**DOI:** 10.1186/1756-8722-4-39

**Published:** 2011-09-27

**Authors:** Hilda Rachel Diamond, Maria Helena Ornellas, Alberto Orfao, Bernadete E Gomes, Mércia M Campos, Teresa S Fernandez, Roberto I da Silva, Gilda Alves, Claudia Lage, Dayse A da Silva, Arthur Moellmann-Coelho, Geydson S da Cruz, Luis Fernando Bouzas, Eliana Abdelhay

**Affiliations:** 1 Laboratory of Immunology, Bone Marrow Transplantation Unit, National Cancer Institute, Praça Cruz Vermelha n° 23, 6° andar. Centro, Rio de Janeiro, RJ, 20230-130, Brazil; 2 Department of Pathology, Rio de Janeiro State University, Avenida Manoel de Abreu 444, 4° andar -Patologia Geral, Rio de Janeiro, RJ, 20550-170, Brazil; 3 Cancer Research Centre (IBMCC-CSIC/USAL), University of Salamanca, Centro de Investigación del Cáncer Paseo de la Universidad de Coimbra s/n37007 Salamanca, Spain; 4 Laboratory of Cytogenetics, Bone Marrow Transplantation Unit, National Cancer Institute, Praça Cruz Vermelha n° 23, 6° andar. Centro, Rio de Janeiro, RJ, 20230-130, Brazil; 5 Laboratory of Applied Genetics, Hematology Service, National Cancer Institute, Praça Cruz Vermelha n° 23, 6° andar. Centro, Rio de Janeiro, RJ, 20230-130, Brazil; 6 Program of Molecular and Structural Biology, Carlos Chagas Filho Biophysics Institute, Rio de Janeiro Federal University, CCS - BLOCO G - SALA G0-031, ILHA DA CIDADE UNIVERSITÁRIA, Rio de Janeiro, RJ, 21941-902, Brazil; 7 Hematology Service, National Cancer Institute, Praça Cruz Vermelha n° 23, 8° andar. Centro, Rio de Janeiro, RJ, 20230-130, Brazil; 8 Clinical Division, Bone Marrow Transplantation Unit, National Cancer Institute, Praça Cruz Vermelha n° 23, 7° andar. Centro, Rio de Janeiro, RJ, 20230-130, Brazil; 9 Stem Cell Laboratory, Bone Marrow Transplantation Unit, National Cancer Institute, Praça Cruz Vermelha n° 23, 6° andar. Centro, Rio de Janeiro, RJ, 20230-130, Brazil

**Keywords:** immunophenotype, cytogenetics, DNA repair, donor origin leukemia

## Abstract

A 54-year-old woman was diagnosed with infiltrative ductal breast carcinoma. Two years after treatment, the patient developed an acute myeloid leukemia (AML) which harbored del(11q23) in 8% of the blast cells. The patient was submitted for allogeneic stem cell transplantation (aSCT) from her HLA-compatible sister. Ten months after transplantation, she relapsed with an AML with basophilic maturation characterized by CD45^low ^CD33^high^, CD117^+^, CD13^-/+^, HLA Dr^high^, CD123^high^, and CD203c^+ ^blast cells lacking expression of CD7, CD10, CD34, CD15, CD14, CD56, CD36, CD64, and cytoplasmic tryptase. Karyotype analysis showed the emergence of a new clone with t(2;14) and FISH analysis indicated the presence of *MLL *gene rearrangement consistent with del(11q23). Interestingly, AML blast cell DNA tested with microsatellite markers showed the same pattern as the donor's, suggesting that this AML emerged from donor cells. Additionally, polymorphisms of the *XPA*, *XPD*, *XRCC1*, *XRCC3 *and *RAD51 *DNA repair genes revealed three unfavorable alleles with low DNA repair capacity.

In summary, we report the first case of AML involving *XPD *and *XRCC3 *polymorphisms from donor origin following allogeneic stem cell transplantation and highlight the potential need for careful analysis of DNA repair gene polymorphisms in selecting candidate donors prior to allogeneic stem cell transplantation.

## Background

Breast cancer is the most frequent malignancy in women [1]. Over recent decades overall survival of breast cancer patients has increased considerably as a result of earlier diagnosis and increasing use of adjuvant therapies [2,3]. Nevertheless, the risk of developing a secondary cancer increases as a long-term complication related to the use of cytotoxic DNA-targeted antiproliferative drugs and hormone therapy with or without radiotherapy [4,5].

Among other complications, a small proportion of all breast cancer survivors subsequently develop acute myeloblastic leukemia (AML), preceded or not by a preleukemic myelodysplastic syndrome (MDS) [5]. Secondary AML has many morphological and cytogenetic variants because transforming mutations leading to the disease are heterogeneous and occur in an early multipotential hematopoietic cell that retains the potential to differentiate into virtually every hematopoietic lineage [6].

Here we report a rare case of a donor-related secondary AML with basophilic maturation post-allogeneic stem cell transplantation in a patient with prior history of secondary AML derived from primary breast cancer chemotherapy. To our knowledge this is the first case reported in the literature of a donor cell-derived AML secondary to breast cancer treatment and allogeneic stem cell transplantation associated with unfavourable DNA repair gene polymorphisms.

## Case presentation

A 54-year-old woman was submitted for mastectomy in May 2004 because of an infiltrative ductal breast carcinoma, with negative nodal infiltration and without expression of hormone receptors. After surgery, she was treated with cyclophosphamide (600 mg/m^2^) and doxorubicin (60 mg/m^2^; 4 cycles) followed by adjuvant radiotherapy. In December 2006, the patient presented with fever, anemia, and gingival bleeding. Peripheral blood data revealed anemia (hemoglobin level of 7.7 g/L) and thrombocytopenia (12 × 10^9 ^platelets/L) with an increased white blood cell count (35.4 × 10^9 ^leucocytes/L), with 70% blasts. A bone marrow aspirate sample showed diffuse infiltration by blast cells and diagnosis of AML M5 according to the French-American-British (FAB) classification was made. On immunophenotypic criteria, blast cells were positive for CD33, CD117, CD13, and HLA-DR.

Cytogenetic studies performed on a bone marrow aspirate sample using standard culture methods and GTG banding revealed a normal 46, XX[25] karyotype at the time of the diagnosis of secondary AML prior to allogeneic stem cell transplantation (allo-SCT). Cytogenetic markers for secondary AML [del(11)(q23), del(5q)/-5 or del(7q)/-7] were further investigated by interphase fluorescence *in situ *hybridization (iFISH) using the LSI *MLL *(11q23) dual color, LSI D7S486 spectrum orange/CEP7 spectrum green, LSI EGR1 spectrum orange/LSID5S23:D5S721 spectrum green, and LSI CSF 1R spectrum orange/LSID5S23:D5S721 spectrum green (Vysis, Abbott Laboratories, USA) iFISH probes and showed 8% cells carrying del(11q23) in the absence of abnormalities of both chromosomes 5 and 7. The patient was treated with AraC and idarrubicin [7-9] and complete remission was attained. Consolidation was performed with cytarabin-arabinose (high dose Ara-C) plus filgrastin, as prophylaxis for leucopenia.

**Figure 1:**
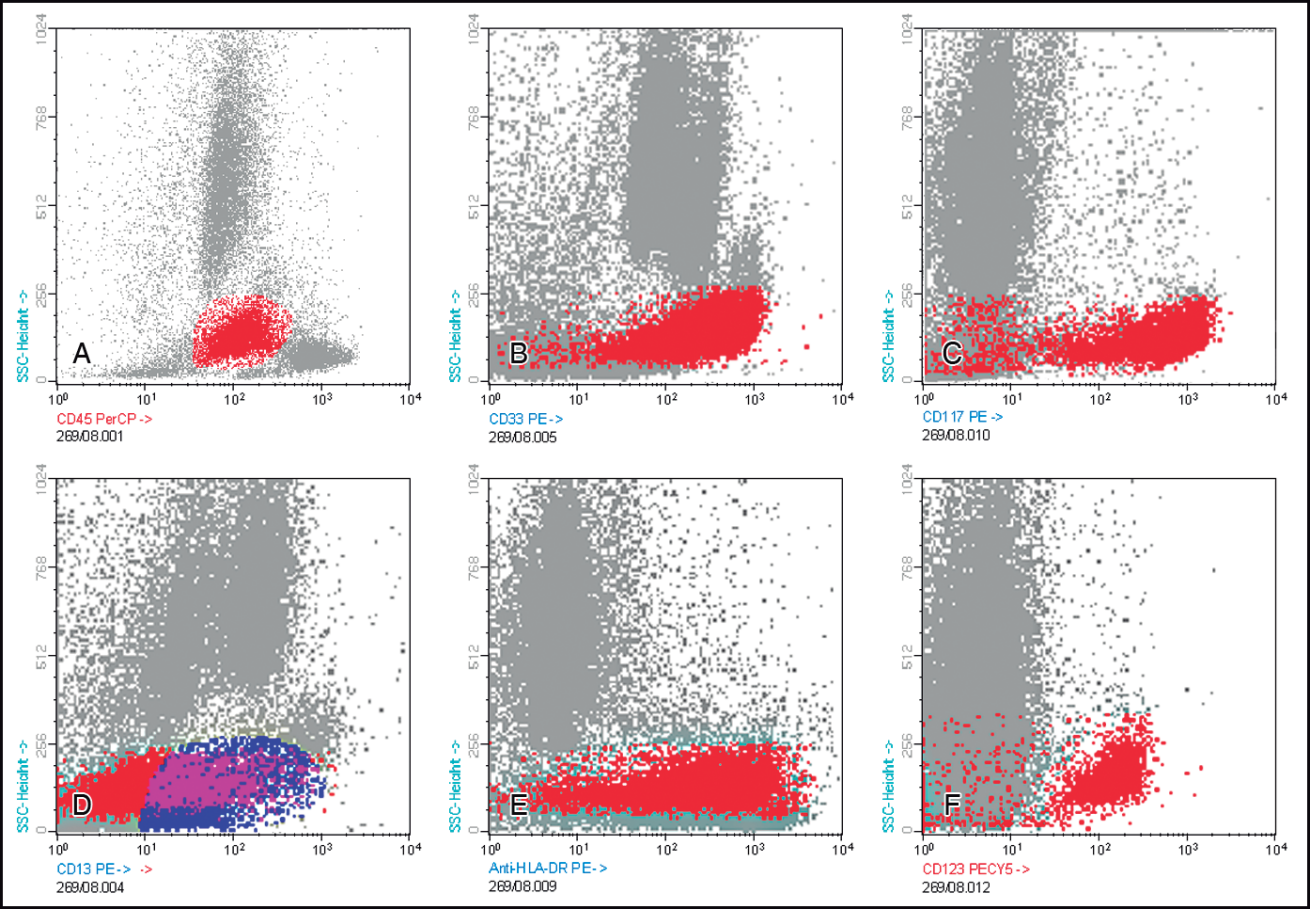
**Bivariate flow cytometry dot plots showing the immunoreactivity pattern of blast cells (gated on CD45 *vs*. SSC) for CD45^low ^= 25% (panel A), CD33 = 24% (panel B), CD117 = 24% (panel C), CD13 = 16% (panel D), HLA-DR = 25% (panel E), and CD123 = 25%(panel F)**.

**Figure 2:**
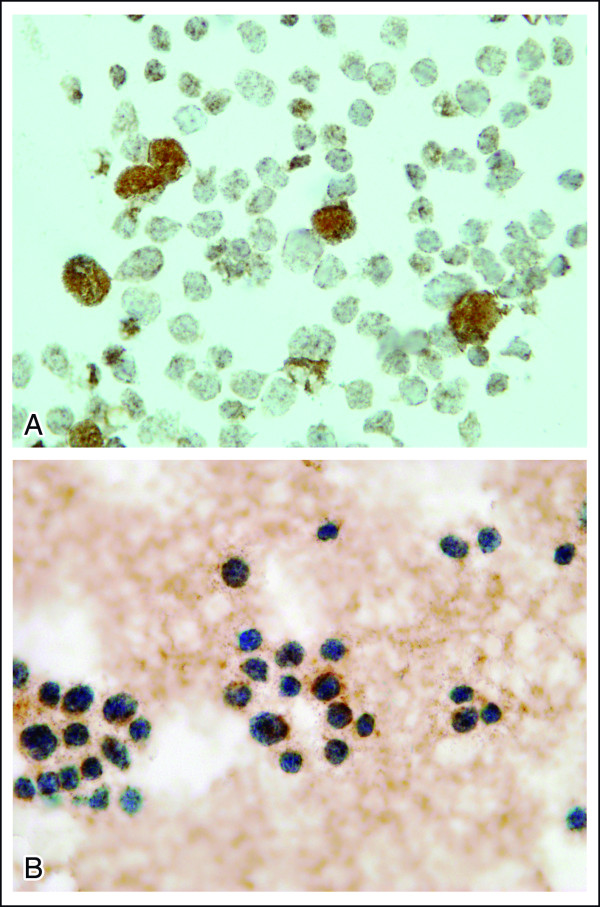
**Bone marrow smear after allo-SCT relapse**. A- Note the presence of basophils with CD203c^+^in the sample with approximately 20% of blasts being CD203c^+^. B- Note the absence of tryptase staining.

After two cycles of consolidation, she was submitted to an allo-SCT from her HLA-compatible sister. The conditioning regimen for the allo-SCT consisted of bussulfan and cyclosphosphamide. At day +48, she developed acute graft versus host disease (aGVHD), and was treated with corticosteroids and cyclosporine (CSA). At day +280, an inguinal 4 × 3 cm mass appeared and the presence of malignant cells was revealed upon biopsy. At day +300, a bone marrow aspirate showed 50% blasts. Cellular and molecular analyses were performed in parallel on this sample. Immunophenotyping of bone marrow cells confirmed the presence of CD45^low ^CD33^high^, CD117^+^, CD13^-/+^, HLA Dr^high ^CD123^high ^blast cells, lacking expression of CD7, CD10, CD34, CD15, CD14, CD56, CD36, and CD64 (Figure 1A-F), suggesting maturation into the basophil *vs*. mast cell lineages. Further immunocytochemical stainings were performed showing CD203c expression in the absence of cytoplasmic tryptase (Figure 2A and 2B); these together with the high expression for CD123 were consistent with basophilic maturation.

Microsatellite and PCR-RFLP analyses were performed on genomic DNA from mononuclear cells of the patient pre-transplant, of the AML blast cell sample obtained after transplantation, and of the donor bone marrow cells. The 11 tested microsatellite markers are often used in forensic medicine for individual identification. As displayed in Table 1, 7 of the analyzed loci (D21S11, D7S820, CSF1PO, D3S1358, Vwa, D13S317, and TPOX) were informative and showed the coincidence of profile between AML blasts after transplantation and the donor's cells, supporting full engraftment of the stem cell transplant as well as the donor cell-origin of the AML blasts.

**Table 1:** Microsatellite markers in the patient's pre and post-transplant haematopoietic cells compared with the donor's

Marker	Patient pre-SCT	Patient AML	Donor
D21S11	28	**28**	**28**
	31.2	**29**	**29**

D7S820	8	**8**	**8**
	10	**9**	**9**

CSF1PO	11	**8**	**8**
	12	**11**	**11**

D3S1358	15	**15**	**15**
	15	**17**	**17**

TH01	8	8	8
	9	9	9

D13S317	9	**11**	**11**
	13	**13**	**13**

D16S539	13	13	13
	14	14	14

Vwa	16	**17**	**17**
	18	**18**	**18**

TPOX	8	**9**	**9**
	11	**12**	**12**

D5S818	12	12	12
	13	13	13

FGA	21	21	21
	23	23	23

Informative and coincident markers are identified in bold.

**Figure 3:**
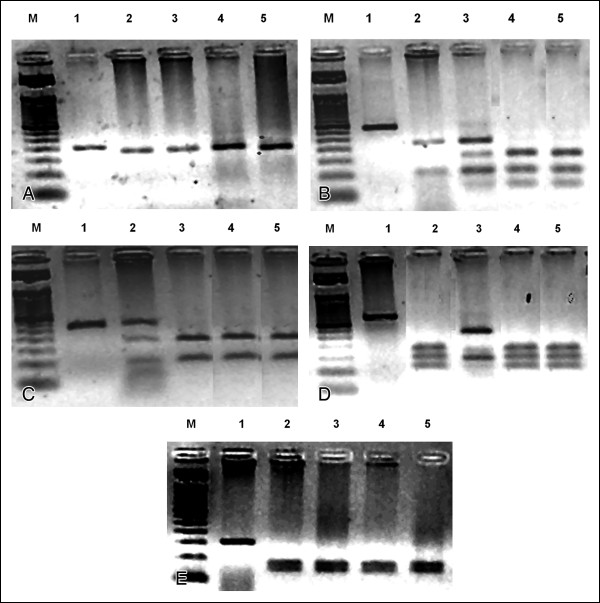
**RFLP analysis of DNA generated by digestion of *XPA *(3A), *XPD *(3B), *XRCC1 *(3C), *XRCC3 *(3D), and *RAD51 *(3E) PCR products digested with or without their specific RFLP diagnostic restriction enzymes**. M = 50 bp ladder marker; Lane 1 = non-digested PCR amplification of normal allele; Lane 2 = digestion pattern from the positive control K562 myeloid cell line alleles; Lane 3 = digestion pattern from patient alleles before transplantation; Lane 4 = digestion pattern from patient alleles from AML cell DNA after transplantation; Lane 5 = digestion pattern from donor cell alleles.

Genetic polymorphisms of five relevant human DNA repair genes (*XPA*, *XPD*, *XRCC1*, *XRCC3*, and *RAD51*) were further analyzed by PCR-RFLP [10-15] on the secondary AML blast DNA and compared to a pre-transplant DNA sample from the patient and to DNA from the donor. Analysis of leukemia-prone polymorphic alleles (*XPA *A23G, *XPD *Lys751Gln, *XRCC1 *Arg399Gln, *XRCC3 *Thr241Met, and *RAD*51 G135C) revealed an *XPD *and *XRCC3*-deficient heterozygous pre-SCT patient with normal alleles for *XPA*, *XRCC1*, and *RAD51 *DNA repair functions (Table 2 and Figure 3). Donor polymorphisms were different, harboring both *XPD*- and *XRCC3*-deficient homozygosis. Once microsatellite analysis indicated post-transplant total chimerism, post-SCT patient cells were shown to have acquired the genotypic markers of the donor's poorer DNA repair functions (Table 2 and Figure 3).

**Table 2:** DNA repair polymorphisms in the patient's pre- and post-transplantation (AML) cells and donor's cells

Polymorphism	DNA repair mechanism	Patient cells (pre-transplantation)	AML cells (post-transplantation)	Donor cells
*XPA *A23G	NER	Homozygous for optimal DRC	Homozygous for optimal DRC	Homozygous for optimal DRC
*XPD *Lys751Gln	NER	Heterozygous deficient DRC	Homozygous for deficient DRC	Homozygous for deficient DRC
*XRCC1 *Ar399Gln	BER	Homozygous for optimal DRC	Homozygous for optimal DRC	Homozygous for optimal DRC
*XRCC3 *Thr243Met	HRR	Heterozygous for deficient DRC	Homozygous for deficient DRC	Homozygous for deficient DRC
*RAD51 *G135C	HRR	Homozygous for optimal DRC	Homozygous for optimal DRC	Homozygous for optimal DRC

DRC - DNA repair capacity

NER - Nucleotide Excision Repair

BER - Base Excision Repair

HRR - Homologous Recombination Repair

**Figure 4:**
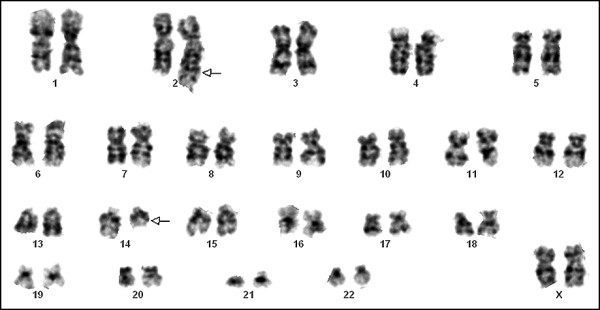
**Illustrating metaphase (G-banding) of AML cells obtained after initial therapy of AML: 46, XX, t(2;14)(q37;q22)[2]/46, XX[33]**. Arrows denote the chromosomes assumed to be involved in t(2;14)(q37;q22).

The patient was then treated with idarubicin, cytosine arabinoside (ARA-C) with no response. At that time the immunophenotypic study showed the same profile, but conventional cytogenetics revealed the emergence of a new clone: 46, XX, t(2;14)(q37;q22) [2]/46, XX [33] (Figure 4). Treatment was modified and FLAG [fludarabin, ARA-C, and granulocyte colony-stimulating factor (G-CSF)] was started. After 18 days the patient had hematopoietic recovery with 3% blasts. Twenty-one days after FLAG, a donor lymphocyte infusion (DLI) was given, which was followed by GVHD, and the patient was treated with corticosteroids. The patient was submitted to a second DLI four months later. At day +14 after this second DLI, the karyotype post-DLI was normal, 46, XX[43], but FISH analysis indicated the presence of *MLL *gene rearrangements consistent with del(11q23) in 5% of the cells. The myelogram showed 62% blasts. Rescue therapy with high dose topotecan and ARA-C was started and administered for five days without haematologic response; peripheral blood infiltration by blast cells rose to 80% after 14 days. Palliative support began and the patient died after 17 months of stem cell infusion.

## Discussions and Conclusions

Here we report a case of a secondary AML developing from donor-derived cells in a breast cancer patient who underwent allogeneic stem cell transplantation. Donor cell leukemia is a rare although well-recognized disease entity following SCT that occurs as the result of oncogenic transformation of apparently normal donor hematopoietic cells in the transplant recipient.

Many studies have reported an increased risk of breast cancer patients to develop leukemia after chemotherapy, radiotherapy and G-CSF administration [1,5,16,17]. Because of this, risk estimates on the eventual development of post-treatment AML/MDS have to be cast when deciding a patient's treatment. In these studies, increased risk of AML/MDS has been reported for cases treated with alkylating agents and anthracyclines. Post-treatment secondary leukemias associated with prior administration of alkylating agents (e.g., cyclophosphamide) typically differ from those arising after treatment with DNA topoisomerase II inhibitors, such as anthracyclines. Accordingly, AML's developing after topoisomerase II inhibitors are given typically show an early onset, and display monocytic and myelomonocytic features in association with abnormalities of chromosomes 11 and 21 (especially balanced translocations involving the 11q23 and 21q22 regions). Whereas those arising after treatment with alkylating agents frequently show neutrophil/granulocytic maturation together with abnormalities of chromosome 5 and 7. These changes are seen in the absence of chromosomal translocations and the leukemias emerge much later after therapy [18].

Exposure to radiotherapy may further increase the risk for AML [19-21]. In turn, Smith *et al. *[20] found the M4/M5 subtypes to be more frequent in patients receiving intense treatment regimens and concluded that this could be the result of cyclophosphamide-induced promotion of a doxorubicin-associated leukemogenic effect. In the case reported here, the association of chemotherapy and radiotherapy protocols most probably played a role in the development of AML. Secondary AML outcomes are thus believed to arise from genomic instability (*i.e.*, deletions, mutations, translocations) induced by therapy-associated DNA damage [22,23]. Two different hypotheses remain which could contribute to explain the development of secondary AML: a truly stochastic event, or individual differences on cancer susceptibility [22]. The latter appears to better explain the case reported here. In this regard, previous reports have described polymorphisms conferring sensitivity to chemotherapy, which may contribute to the incidence of secondary AML outcomes [24-26]. Alternatively, it is possible that germline variations in DNA repair genes may also enhance the risk of therapy-induced secondary AML in patients carrying DNA-repair deficient genes [27]. In fact it has also been shown that single nucleotide polymorphisms (SNP) in DNA repair genes may code malfunctioning proteins, in association with an increased predisposition to cancer and the response of leukemia patients following chemotherapy [12,28,29]. Here we investigated five alleles of DNA repair genes already ascribed to susceptibility to leukemia (*XRCC1*, *XPA*, *XPD*, *XRCC3 *and *RAD51*). As expected, polymorphic alleles found by genotyping in both the patient post-SCT hematopoietic cells and the donor's cells were the same, revealing successful transplant, which could also explain the otherwise observed resistance to treatment and clinical evolution. In turn, pre-SCT patient cells were heterozygous for allelic variants in the *XPD *and *XRCC3 *genes that are associated with lower repair capacity, conspicuously bearing on susceptibility to breast cancer and chemotherapy-related leukemia (AML) in this patient. Consistent with our observations, Allan *et al. *[12] showed that individuals carrying at least one *XPD*- Lys751Gln allele were more likely to have an adverse prognosis following chemotherapy. The *XPD *Gln751 polymorphic protein was shown to fail in engaging apoptosis in chemotherapy-damaged cells, thus avoiding elimination of mutated myeloid precursors. In order to search for an association of the poor outcome of the patient's secondary post-SCT leukemia, the same gene set of polymorphisms was screened for in the donor's DNA. Remarkably, donor DNA genotyped homozygous not only for the *XPD *(Lys751Gln) suboptimal allele, but also for the *XRCC3*-deficient allele (Thr243Met). Both post-SCT blast cells and donor DNA were coincident in *XPD*- and *XRCC3*-deficient homozygosity, supporting a donor origin for the leukemic blasts.

The *XRCC3 *protein plays a critical role in Homologous Recombination Repair (HRR) accounting for repair of DNA double-strand breaks (DSB). Whenever it is deficient, unsealed or misrepaired breaks can generate oncogenic chromosomal translocations. When found together, allelic variants in Lys751Gln *XPD *and Thr241Met *XRCC3 *polymorphisms have been associated with both a worse repair capacity and resistance to apoptosis [11,30]. Once introduced into the patient's drug-intoxicated bone marrow, disrupted DNA repair and apoptosis pathways in the donor's cells may have accumulated unrepaired damages while escaping apoptosis, thus boosting aggressiveness. Indeed, this is further supported by the observation of a switch from normal to highly abnormal post-SCT of the patient's karyotype and FISH analysis. This supports the theory that in donor origin leukemia, the host environment in which the original malignancy developed could trigger an oncogenic process in donor cells, favored by the immunosuppressive status after transplantation, especially because the donor is still healthy [31,32].

An additional interesting finding is the maturation observed phenotypically towards the basophilic versus mast cell lineages based on coexpression of CD203c and both CD123, CD117 [33]. Cases of *de novo *AML with predominant basophilic and/or mastocytic, cell phenotypes are uncommon and they account for only 4-5% of all cases of acute nonlymphocytic leukemia [34,35]. Although acute basophilic leukemia (ABL) has been long diagnosed as such, current knowledge of this specific AML subtype remains limited [36].

In conclusion, here we describe a very rare case of donor origin AML from a sibling who harbored germline *XPD *and *XRCC3 *homozygous polymorphisms in a breast cancer patient following chemotherapy. The blasts showed SNP profiles of the donor including susceptible alleles for unfavorable genotypes in DNA repair genes. Determination of the donor's DNA repair genotype, capable of stimulating genetic instability in a diseased recipient, could be important for future transplantation procedures and therefore should be investigated further. DNA repair mechanisms are responsible for maintenance of the genome integrity avoiding additional mutations in key cell cycle regulation genes which are responsable for "leukemization". To ensure that DNA repair mechanisms are properly working, more donor cell gene polymorphisms or mutations should be studied. After the interpretation of results, a reference panel of low DNA repair capacity polymorphisms or mutations should be included in the international guidelines for screening donor DNA before SCT.

## Consent

The Ethics Committee of the National Cancer Institute performed in accordance with the ethical standards laid down in the 1964 Declaration of Helsinki, approved this study (# of registration 41/11). The written informed consent was obtained from the sister's patient.

A copy of the written consent is available for review by the Editor-in-Chief of this journal.

## Competing interests

The authors declare that they have no competing interests.

## Authors' contributions

HRD, designed the paper and wrote the paper. AO, performed immunocytochemical stainings and reviewed the manuscript. BEG and MMC, performed flow cytometric immunophenotyping. TSF performed cytogenetic and FISH analysis. RIS, GA, CL and DAS, performed the molecular biology studies. AM-C and GSC, were responsible of the patient's treatment and conceived the study. GSC and MHO, carried out acquisition of data's patient. CL, GA and MHO were responsible for manuscript review. EA and LFB carried out their critical interpretations. All authors read and approved the final manuscript.
